# Correction: Adult outcomes by parental, school and postcode aggregated income in childhood—A descriptive analysis of the cohorts 1981–1989 in Finland

**DOI:** 10.1371/journal.pone.0337956

**Published:** 2025-12-02

**Authors:** Aapo Hiilamo, Niilo Luotonen, Lauri Mäkinen, Tiina Ristikari

In the Abstract, there is an error in the seventh sentence of the paragraph. The correct sentence is: People with a lower parental, school, or postcode income were more likely to have children at age 30. At the same time, people with low parental income were less likely to live with a partner.

In the Discussion, there is an error in the third and fourth sentence of the sixth paragraph. The correct sentence is: In cohorts born before 1985, parental income was not consistently linked to partnership status. But a stronger association, showing a higher prevalence of cohabitation or marriage among people with middle- and high-income parents, emerged in the more recent cohorts because the decline in cohabitation or marriage was more rapid among those with low parental income.

## References

[1] Hiilamo A, Luotonen N, Mäkinen L, Ristikari T. Adult outcomes by parental, school and postcode aggregated income in childhood-A descriptive analysis of the cohorts 1981-1989 in Finland. PLoS One. 2025;20(7):e0327364. doi: 10.1371/journal.pone.0327364 40663534 PMC12262847

