# Supplementary material for: Adult outcomes by parental, school and postcode aggregated income in childhood—A descriptive analysis of the cohorts 1981–1989 in Finland
Source: PLoS One. 2025 Jul 15;20(7):e0327364. doi: 10.1371/journal.pone.0327364 (PMC12262847; doi:10.1371/journal.pone.0327364)
Supplement: S1 Fig — The figure plots the unemployment rate the year each of the studied cohorts (born 1981–1989) turned 30. Source: Statistics Finland. https://pxdata.stat.fi/PxWeb/pxweb/fi/StatFin/StatFin__tyti/statfin_tyti_pxt_13ak.px/table/tableViewLayout1/. (DOCX) [file pone.0327364.s001.docx]

**S1 Fig. The Finnish unemployment rate during the sample period. The figure plots the unemployment rate the year each of the studied cohorts (born 1981–1989) turned 30. Source: Statistics Finland.** [**https://pxdata.stat.fi/PxWeb/pxweb/fi/StatFin/StatFin__tyti/statfin_tyti_pxt_13ak.px/table/tableViewLayout1/**](https://pxdata.stat.fi/PxWeb/pxweb/fi/StatFin/StatFin__tyti/statfin_tyti_pxt_13ak.px/table/tableViewLayout1/)
