# Supplementary material for: Adult outcomes by parental, school and postcode aggregated income in childhood—A descriptive analysis of the cohorts 1981–1989 in Finland
Source: PLoS One. 2025 Jul 15;20(7):e0327364. doi: 10.1371/journal.pone.0327364 (PMC12262847; doi:10.1371/journal.pone.0327364)
Supplement: S1 Table — (DOCX) [file pone.0327364.s002.docx]

S1 Table. Family Income 1^st^ and 99^th^ percentile range by Decile and Year

| decile | 1981 | 1982 | 1983 | 1984 | 1985 | 1986 | 1987 | 1988 | 1989 |
| --- | --- | --- | --- | --- | --- | --- | --- | --- | --- |
| 1 | 1650 - 10050 | 1750 - 10450 | 1800 - 10650 | 1650 - 10950 | 2100 - 11400 | 1600 - 12050 | 2100 - 12650 | 2050 - 13200 | 2750 - 13800 |
| 2 | 10100 - 11850 | 10500 - 12350 | 10700 - 12700 | 11000 - 13050 | 11500 - 13650 | 12100 - 14400 | 12700 - 15150 | 13250 - 15750 | 13900 - 16550 |
| 3 | 11850 - 13100 | 12400 - 13700 | 12750 - 14150 | 13100 - 14600 | 13700 - 15300 | 14450 - 16100 | 15200 - 16850 | 15800 - 17550 | 16600 - 18400 |
| 4 | 13150 - 14150 | 13750 - 14800 | 14200 - 15300 | 14600 - 15800 | 15300 - 16550 | 16100 - 17450 | 16900 - 18250 | 17600 - 19000 | 18450 - 19950 |
| 5 | 14200 - 15150 | 14850 - 15900 | 15350 - 16400 | 15800 - 17000 | 16600 - 17750 | 17500 - 18700 | 18300 - 19600 | 19000 - 20400 | 20000 - 21400 |
| 6 | 15200 - 16200 | 15900 - 17000 | 16450 - 17600 | 17000 - 18200 | 17750 - 19000 | 18750 - 20100 | 19600 - 21000 | 20400 - 21800 | 21400 - 22900 |
| 7 | 16250 - 17500 | 17050 - 18350 | 17600 - 19000 | 18200 - 19700 | 19050 - 20600 | 20100 - 21750 | 21050 - 22750 | 21850 - 23600 | 22950 - 24850 |
| 8 | 17500 - 19200 | 18400 - 20200 | 19050 - 20950 | 19700 - 21800 | 20600 - 22800 | 21750 - 24100 | 22800 - 25200 | 23650 - 26200 | 24900 - 27700 |
| 9 | 19250 - 22300 | 20300 - 23700 | 21000 - 24700 | 21850 - 25900 | 22850 - 27100 | 24150 - 28500 | 25300 - 29950 | 26250 - 31250 | 27750 - 33350 |
| 10 | 22400 - 43100 | 23850 - 49600 | 24800 - 54800 | 26000 - 63200 | 27250 - 70100 | 28650 - 67300 | 30150 - 69900 | 31450 - 74400 | 33500 - 80600 |
