# Supplementary material for: Adult outcomes by parental, school and postcode aggregated income in childhood—A descriptive analysis of the cohorts 1981–1989 in Finland
Source: PLoS One. 2025 Jul 15;20(7):e0327364. doi: 10.1371/journal.pone.0327364 (PMC12262847; doi:10.1371/journal.pone.0327364)
Supplement: S2 Table — (DOCX) [file pone.0327364.s003.docx]

S2 Table. School Income 1^st^ and 99^th^ percentile Range by Decile and Year

| decile | 1981 | 1982 | 1983 | 1984 | 1985 | 1986 | 1987 | 1988 | 1989 |
| --- | --- | --- | --- | --- | --- | --- | --- | --- | --- |
| 1 | 10650 - 13500 | 11350 - 14200 | 11200 - 14675 | 11500 - 15125 | 11900 - 15825 | 12375 - 16700 | 13200 - 17350 | 14300 - 18200 | 14800 - 19100 |
| 2 | 13550 - 14100 | 14250 - 14800 | 14700 - 15300 | 15150 - 15650 | 15875 - 16500 | 16750 - 17400 | 17400 - 18250 | 18250 - 19000 | 19150 - 19925 |
| 3 | 14125 - 14450 | 14825 - 15200 | 15325 - 15700 | 15675 - 16100 | 16525 - 17000 | 17425 - 17850 | 18300 - 18700 | 19025 - 19500 | 19950 - 20475 |
| 4 | 14475 - 14800 | 15225 - 15550 | 15725 - 16000 | 16125 - 16550 | 17025 - 17375 | 17900 - 18250 | 18750 - 19100 | 19525 - 19900 | 20500 - 20900 |
| 5 | 14850 - 15125 | 15575 - 15850 | 16025 - 16350 | 16600 - 16900 | 17400 - 17700 | 18275 - 18625 | 19125 - 19425 | 19925 - 20300 | 20925 - 21225 |
| 6 | 15150 - 15475 | 15875 - 16150 | 16375 - 16725 | 16925 - 17300 | 17725 - 18000 | 18650 - 19000 | 19450 - 20000 | 20350 - 20750 | 21250 - 21750 |
| 7 | 15500 - 15800 | 16200 - 16525 | 16750 - 17200 | 17325 - 17725 | 18025 - 18400 | 19025 - 19500 | 20025 - 20450 | 20775 - 21225 | 21775 - 22300 |
| 8 | 15850 - 16300 | 16550 - 17075 | 17225 - 17675 | 17750 - 18350 | 18425 - 19000 | 19525 - 20225 | 20475 - 21150 | 21250 - 21800 | 22325 - 22900 |
| 9 | 16325 - 17200 | 17100 - 18050 | 17700 - 18550 | 18375 - 19600 | 19050 - 20175 | 20250 - 21650 | 21175 - 22350 | 21850 - 23075 | 22925 - 24275 |
| 10 | 17250 - 23300 | 18100 - 26000 | 18600 - 26700 | 19700 - 29050 | 20200 - 30650 | 21700 - 30150 | 22500 - 35825 | 23100 - 35750 | 24300 - 35000 |
