# Supplementary material for: Adult outcomes by parental, school and postcode aggregated income in childhood—A descriptive analysis of the cohorts 1981–1989 in Finland
Source: PLoS One. 2025 Jul 15;20(7):e0327364. doi: 10.1371/journal.pone.0327364 (PMC12262847; doi:10.1371/journal.pone.0327364)
Supplement: S3 Table — (DOCX) [file pone.0327364.s004.docx]

S3 Table. Postcode Income 1^st^ and 99^th^ percentile Range by Decile and Year

| decile | 1981 | 1982 | 1983 | 1984 | 1985 | 1986 | 1987 | 1988 | 1989 |
| --- | --- | --- | --- | --- | --- | --- | --- | --- | --- |
| 1 | 7500 - 9500 | 7700 - 9800 | 7800 - 10000 | 8100 - 10300 | 8400 - 10700 | 8850 - 11400 | 9400 - 12000 | 9600 - 12400 | 9900 - 12900 |
| 2 | 9600 - 10100 | 9850 - 10500 | 10100 - 10800 | 10350 - 11000 | 10750 - 11500 | 11500 - 12200 | 12050 - 12800 | 12500 - 13250 | 13000 - 13800 |
| 3 | 10200 - 10600 | 10600 - 11000 | 10900 - 11300 | 11100 - 11600 | 11550 - 12100 | 12250 - 12900 | 12900 - 13500 | 13300 - 14000 | 13900 - 14600 |
| 4 | 10650 - 11000 | 11100 - 11400 | 11400 - 11750 | 11650 - 12100 | 12200 - 12600 | 13000 - 13400 | 13550 - 14100 | 14100 - 14600 | 14700 - 15200 |
| 5 | 11100 - 11400 | 11500 - 11900 | 11800 - 12300 | 12150 - 12600 | 12650 - 13200 | 13450 - 14050 | 14200 - 14700 | 14650 - 15200 | 15300 - 15900 |
| 6 | 11500 - 11800 | 12000 - 12300 | 12400 - 12800 | 12700 - 13200 | 13300 - 13800 | 14100 - 14600 | 14800 - 15300 | 15300 - 15900 | 16000 - 16700 |
| 7 | 11900 - 12300 | 12400 - 12900 | 12850 - 13300 | 13300 - 13800 | 13900 - 14500 | 14700 - 15300 | 15400 - 16000 | 15950 - 16600 | 16800 - 17400 |
| 8 | 12350 - 12800 | 12950 - 13500 | 13400 - 14000 | 13900 - 14500 | 14550 - 15200 | 15350 - 16100 | 16100 - 16800 | 16650 - 17400 | 17500 - 18200 |
| 9 | 12900 - 13500 | 13600 - 14200 | 14050 - 14700 | 14600 - 15400 | 15300 - 16100 | 16150 - 17200 | 16900 - 17800 | 17500 - 18400 | 18300 - 19200 |
| 10 | 13600 - 17000 | 14300 - 19400 | 14800 - 20100 | 15500 - 22000 | 16200 - 23100 | 17300 - 22500 | 17900 - 24600 | 18500 - 24300 | 19300 - 25400 |
