# Supplementary material for: Adult outcomes by parental, school and postcode aggregated income in childhood—A descriptive analysis of the cohorts 1981–1989 in Finland
Source: PLoS One. 2025 Jul 15;20(7):e0327364. doi: 10.1371/journal.pone.0327364 (PMC12262847; doi:10.1371/journal.pone.0327364)
Supplement: Attachment — Submitted filename: response letter.docx [file pone.0327364.s008.docx]

Reviewer #2: Thank you for inviting my review of the revised manuscript by Hiilamo and colleagues on childhood socioeconomic environment and health outcomes in Finland – the manuscript is strengthened with the edits made by the researchers.

The inclusion of information on cohort denominators, numbers of outcome events and data flow shows this is a reasonably large study with few exclusions due to missing information, which is encouraging. Further, the addition of confidence intervals helps with interpreting the results and examining strength of the conclusions made.

I have just one remaining comment, relating to the information on the income bands in each decile which has been added. This tabulation very interestingly shows there is variation over birth cohorts in the income ranges across deciles i.e. what constituted income decile 1 in 1986 (range 1650 - 10050), differs to decile 1 in 1989 (range 2750 - 13800), similarly decile 10 in 1986 (range 22400 - 43100) versus decile 10 in 1989 (range 33500 - 80600). It would appear to me that income has broadly increased over time, and the gap between the decile 1 and 10 has widened. This sounds entirely plausible, however I would ask the authors to consider whether the underlying changes in the distribution of income over time could potentially influence the result in anyway i.e. if it may have exaggerated any of the risk differences or if it is unlikely to have made a material difference.

Response #1:

**Thank you for your assessment and this excellent point. Indeed, during the study period income inequality increased in Finland by several measures including the GINI index (from 0.22 in 1996 to 0.28 in 2004 and the ratio of incomes of the top and last deciles of income distribution (3.1 to 4.1) according to the Statistics of Finland. As the reviewer correctly points out these are reflected in our supplementary table. We agree that this increase in income inequality may have strengthened some of the observed trends.**

**We now write this in the discussion section:**

***“This study remains agnostic about the potential drivers of the observed trends. However, one potential driver of the increasing differences by childhood income in some outcomes may be the growing income inequality in the country. During the study period, income inequality increased in Finland according to several measures, including the Gini index (from 0.22 in 1996, when income was measured for the 1981 cohort, to 0.28 in 2004), and the ratio of incomes between the highest and lowest deciles (from 3.1 to 4.1), according to Statistics Finland [42]. These changes are reflected in Supplementary Table 1, which shows that the differences in income bands between the first and last deciles increased across the cohorts.”***

Other Minor - Table 1. Perhaps add a final row in the table with the total

**Response #2:**

**Thank you! We agree with the reviewer and have added the total row.**
